# Mast Cells, Angiogenesis and Lymphangiogenesis in Human Gastric Cancer

**DOI:** 10.3390/ijms20092106

**Published:** 2019-04-29

**Authors:** Giuseppe Sammarco, Gilda Varricchi, Valentina Ferraro, Michele Ammendola, Michele De Fazio, Donato Francesco Altomare, Maria Luposella, Lorenza Maltese, Giuseppe Currò, Gianni Marone, Girolamo Ranieri, Riccardo Memeo

**Affiliations:** 1 Department of Health Science, General Surgery, Magna Graecia University, Medicine School of Germaneto, 88100 Catanzaro, Italy; sammarco@unicz.it (G.S.); currog@unicz.it (G.C.); 2 Department of Translational Medical Sciences (DISMET) and Center for Basic and Clinical Immunology Research (CISI), University of Naples Federico II, 80131 Naples, Italy; gildanet@gmail.com (G.V.); marone@unina.it (G.M.); 3 WAO Center of Excellence, 80131 Naples, Italy; 4 Department of Biomedical Sciences and Human Oncology, Unit of Endocrine, Digestive and Emergency Surgery, Aldo Moro University, 74124 Bari, Italy; ferrarov.v@libero.it; 5 Department of Emergency and Organ Transplantation, Aldo Moro University, 74124 Bari, Italy; micheledefazio@uniba.it (M.D.F.); donatofrancesco.altomare@uniba.it (D.F.A.); 6 Cardiovascular Disease Unit, San Giovanni di Dio Hospital, 88900 Crotone, Italy; marilyn_luposella@live.it; 7 Pathology Unit, Pugliese-Ciaccio Hospital, 88100 Catanzaro, Italy; lorenza.maltese@alice.it; 8 Department of Human Pathology of Adult and Evolutive Age G. Barresi, University of Messina, 98122 Messina, Italy; 9 Institute of Experimental Endocrinology and Oncology (IEOS), National Research Council (CNR), 80131 Naples, Italy; 10 Interventional Oncology Unit with Integrated Section of Translational Medical Oncology, National Cancer Research Centre, Istituto Tumori Giovanni Paolo II, 74124 Bari, Italy; giroran@tiscalinet.it

**Keywords:** angiogenesis, cancer, gastric cancer, immune cells, inflammation, lymphangiogenesis, mast cells

## Abstract

Gastric cancer is diagnosed in nearly one million new patients each year and it remains the second leading cause of cancer-related deaths worldwide. Although gastric cancer represents a heterogeneous group of diseases, chronic inflammation has been shown to play a role in tumorigenesis. Cancer development is a multistep process characterized by genetic and epigenetic alterations during tumour initiation and progression. The stromal microenvironment is important in maintaining normal tissue homeostasis or promoting tumour development. A plethora of immune cells (i.e., lymphocytes, macrophages, mast cells, monocytes, myeloid-derived suppressor cells, Treg cells, dendritic cells, neutrophils, eosinophils, natural killer (NK) and natural killer T (NKT) cells) are components of gastric cancer microenvironment. Mast cell density is increased in gastric cancer and there is a correlation with angiogenesis, the number of metastatic lymph nodes and the survival of these patients. Mast cells exert a protumorigenic role in gastric cancer through the release of angiogenic (VEGF-A, CXCL8, MMP-9) and lymphangiogenic factors (VEGF-C and VEGF-F). Gastric mast cells express the programmed death ligands (PD-L1 and PD-L2) which are relevant as immune checkpoints in cancer. Several clinical undergoing trials targeting immune checkpoints could be an innovative therapeutic strategy in gastric cancer. Elucidation of the role of subsets of mast cells in different human gastric cancers will demand studies of increasing complexity beyond those assessing merely mast cell density and microlocalization.

## 1. Introduction

Gastric cancer is the fourth-most-common cancer globally and the second-leading cause of cancer deaths [1,2,3]. It accounts for nearly one million cases annually, with East Asia accounting for more than half of those cases [1,4]. In addition to incidence, the clinicopathologic characteristics of gastric cancer also differ among regions, especially Asia and the West [4,5]. Compelling evidence indicates that gastric cancer is a heterogeneous disease [6,7,8,9] on the basis of anatomic site [10], histopathology [11], gene expression [12,13,14,15,16], gene amplification [13,17], DNA methylation [13,18,19,20], relevant genetic aberrations [13,17,21,22,23,24] and oncogenic pathways [13,25,26].

Cancer development is a multistep process characterized by genomic instability, gene expression dysregulation and epigenetic abnormality that drive tumour progression [27]. Gene mutations and mutant cells are constantly generated but the immunosurveillance system detects and eliminates these cells [28]. However, immune-resistant cells evolve sophisticated strategies to evade the immune system and go on to generate tumours. Angiogenesis, the formation of new blood vessels, is essential for tumour growth, whereas lymphangiogenesis, the development of new lymphatic vessels, is important in the formation of metastases [29,30].

The stromal microenvironment plays a major role in maintaining normal tissue homoeostasis or promoting tumour growth. Mounting evidence indicates that normal tissue microenvironment is a barrier to tumorigenesis, whereas incorrect proinflammatory signals (e.g., cytokines, chemokines, reactive oxygen species, low pH, hypoxia, adenosine, etc.) destabilize tissue homeostasis and promote tumorigenesis [31]. Prolonged and uncontrolled low-grade inflammation or smouldering inflammation is a hallmark of cancer and several immune cells (macrophages, mast cells, lymphocytes, neutrophils, NK and NKT cells, etc.) are stromal components of the inflammatory microenvironment that modulates the development of experimental and human tumours [32,33,34,35].

Mast cells are immune cells present in all classes of vertebrates [36] which were identified in human tumour and named by Paul Ehrlich [37]. These cells have a widespread distribution in close proximity to epithelia, fibroblasts, blood and lymphatic vessels and nerves [38]. Human mast cells form a highly heterogeneous population of cells with different morphology, mediators and surface receptors [39]. These cells derive from CD34^+^, CD117^+^ (KIT) pluripotent hematopoietic stem cells in the bone marrow [40]. Mast cell progenitors enter the circulation and complete their maturation in tissues. These cells are involved in several physiological and inflammatory processes, including organ development [41], skin barrier homeostasis [42], angiogenesis [43], lymphangiogenesis [44], wound healing [45], heart function [46,47], coagulation [48] and tumorigenesis [35,49,50,51,52,53,54,55].

Mast cells have the capacity to rapidly perceive metabolic and immunologic insults and initiate different biochemical programs of homeostasis or inflammation. These cells are activated not only by IgE [56], specific antigens [57] and superantigens [58,59], the mechanisms which account for their functions in allergic diseases but also by a plethora of immunologic and non-immunologic stimuli [60,61,62,63]. 

Mast cell activation leads to the release of a large repertoire of biologically active mediators that have potential positive or negative effects on various targets [30,64,65]. Mast cell mediators have been canonically associated with a detrimental role in allergic diseases [38,39,57,66]. Given their presence in nearly all tissues and the plethora of proinflammatory and immunoregulatory mediators they produce and their capacity to interact closely with several immune and non-immune cells, mast cells are involved in several pathophysiological processes [67]. 

## 2. Mast Cells and Tumour Biology

Mast cells in human tumour were initially described by Paul Ehrlich and extended by Eugen Westphal [68]. Tumour-associated mast cells (TAMCs) are a component of the microenvironment of nearly all solid [49,69,70,71,72,73,74,75,76,77,78,79,80,81,82] and haematologic human tumours [83,84,85,86,87,88,89,90,91,92,93]. TAMCs may exert pro- or anti-tumorigenic roles depending on but not limited to, the tumour model, the stage and the type of tumour and their localization within the tumour (i.e., intra-tumoral vs peri-tumoral) [35,94]. In a few cases, they appear to be inert bystanders [95,96,97,98]. Recent evidence indicates that mast cells [99,100], like macrophages [101,102,103] and neutrophils [104,105,106] are heterogeneous. 

Mast cells are recruited into tumour microenvironment (TME) by several tumour cell-derived chemotactic factors. For example, stem cell factor (SCF) acts on the mast cell KIT receptor [62,107], vascular endothelial growth factors (VEGFs) act on VEGFR1 and VEGFR2 [44,79], angiopoietin 1 (ANGPT1) acts on TIE2 receptor [108] and CXCL8 acts on CXCR1 and CXCR2 [109]. Several chemokines (CCL2, CCL5, CXCL1, CXCL10 and CXCL12) produced by tumour and stromal cells activate their specific mast cell receptors (CCR2, CCR3, CXCR2, CXCR3 and CXCR4), which are important for TAMC localization in TME [49,79,110,111,112,113,114,115,116]. 

Histamine, a major proinflammatory mediator released by activated mast cells, exerts a paracrine chemotactic effect through the engagement of histamine H_4_ receptor on mast cells [117]. PGE_2_, produced by several tumours, is chemotactic for mast cells through the activation of EP3 receptor [118]. Finally, osteopontin, which is upregulated in human cancer [49], affects mast cell migration [119].

Within the tumour microenvironment, TAMCs are exposed to and activated by several factors. Adenosine, produced by tumour cells and mast cells [120], is markedly increased in the TME [121,122] and potentiates the production of angiogenic factors from human mast cells and macrophages [109,123,124]. Hypoxia, a prominent feature of TME [121], activates human mast cells to release IL-6 [125] and VEGF-A [126]. Cyclooxygenase 2 (COX-2), overexpressed in tumours, [121] produces PGE_2_ which fosters angiogenic and lymphangiogenic factors from human mast cells [44]. Several chemokines (i.e., CXCL1, CXCL10, CXCL12) activate mast cells and enhance mast cell secretion of CXCL8 [79,115] which promotes epithelial-to-mesenchymal transition of cancer cells [109,124]. Increased expression of immunoglobulin free light chains (FLCs) was found in various human cancers, activates mast cells [127,128] and promotes tumour growth in a murine B16-F10 melanoma model [61]. Gastric cancer-derived adrenomedullin induced mast cell degranulation [129].

TAMCs modulate recruitment and activation of other immune cells at tumour sites. For example, TAMCs mobilize myeloid-derived suppressor cells (MDSCs) that foster tumour growth owing through their immunosuppressive properties [130]. Moreover, mast cells enhance MDSCs functions in vitro and in vivo [131,132,133,134]. 

## 3. Mast Cells in Tumour Angiogenesis and Lymphangiogenesis

Angiogenesis and lymphangiogenesis occur vigorously during embryogenesis but are restricted during adulthood [135]. In adults, angiogenesis and lymphangiogenesis are limited to sites of wound healing [136] and inflammation [137]. Angiogenesis is a hallmark of cancer because its induction is indispensable to fuel tumour growth [138]. Several innate immune cells can drive angiogenesis during tumour growth, primarily through the production of angiogenic molecules within the TME [65]. Tumour lymphangiogenesis may occur both within the primary tumour and/or in the tumour periphery [139] and plays a central role in the formation of metastasis [139,140]. Angiogenesis and lymphangiogenesis are controlled by stimulatory and inhibitory signals [29,135,141]. VEGF-A is a potent agonist of vascular endothelial growth factor receptor 2 (VEGFR2) on blood endothelial cells (BECs) [142]. VEGF-C and VEGF-D are crucial for the survival, proliferation and migration of lymphatic endothelial cells (LECs) [143] through the engagement of VEGFR3 [144].

VEGF-A, VEGF-B, VEGF-C, VEGF-D and placenta growth factor (PlGF) bind to three endothelial receptors: VEGFR1, VEGFR2 and VEFGR3 [145]. VEGF-A induces the survival, proliferation, sprouting and migration of BECs, increases endothelial permeability [146,147] and promotes inflammation [44,148,149]. VEGF-A also modulates lymphangiogenesis by binding to VEGFR2/VEGFR3 heterodimer receptor [142] and indirectly by recruiting immune cells (e.g., macrophages, mast cells) that produce VEGF-C and VEGF-D [44,150]. PlGF and VEGF-B bind to VEGFR1 on BECs [151], some immune cells and pericytes [148,149,152]. Angiopoietins (ANGPT1 and ANGPT2) modulate angiogenesis and lymphangiogenesis [153] through the engagement of TIE1 and TIE2 receptors [154]. ANGPT1 expressed by pericytes fosters BEC survival, whereas ANGPT2, secreted by BECs, acts autocrinally and paracrinally as TIE2 ligand [153]. Human lung mast cells express TIE1 and TIE2 and ANGPT1 induces migration of these cells by binding to TIE2 [108]. Certain chemokines also modulate angiogenesis and lymphangiogenesis [155,156].

**Figure 1:**
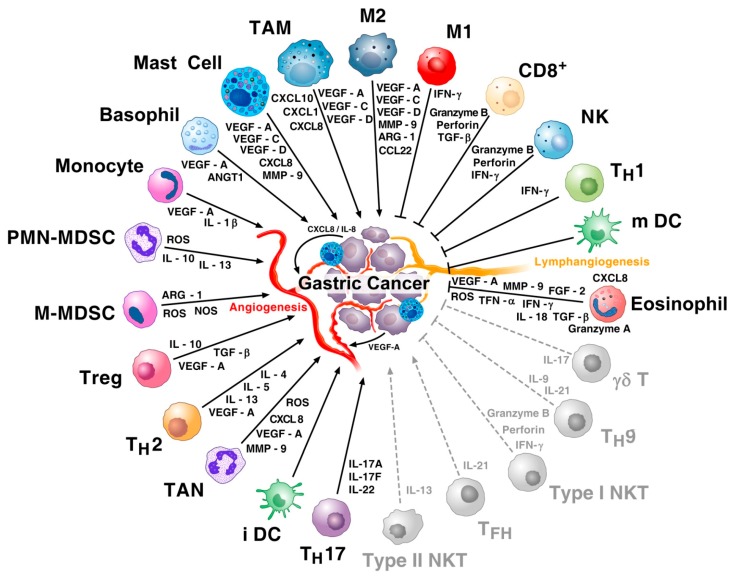
Representation of the immune landscape of human gastric cancer. The immune network in gastric cancer is a complex and dynamic system characterized by multiple interactions between a wide spectrum of immune cells, their mediators and tumour cells. Tumour-associated macrophages (TAM), M2 macrophages, tumour-associated mast cells, basophils, monocytes, polymorphonuclear-myeloid-derived suppressor cells (PMN-MDSCs), monocyte-derived suppressor cells (M-MDSCs), Tregs, Th2 cells, tumour-associated neutrophils (TAN), immature DCs (iDCs), Th17 cells and their mediators play protumorigenic roles. M1 macrophages, cytotoxic CD8^+^ T cells, NK cells, Th1 cells, mature DCs (mDCs) and their mediators play an anti-tumorigenic role in gastric cancer. VEGF-A and CXCL8 produced by tumour cells can activate tumour angiogenesis. Mast cells and macrophages are major producers of lymphangiogenic factors (VEGF-C and VEGF-D). The anti-tumorigenic role of Th9 cells, type I NKT cells and γδ T cells (grey and dashed lines) have been demonstrated in several other human cancers or are under investigation in gastric cancer. There is increasing evidence that eosinophils play an anti-tumorigenic role in different cancers [162,163,164]. The protumorigenic role of circulating Tfh cells [165] and of type II NKT cells has been preliminarily shown in gastric cancer or in several other human tumours, respectively (grey and dashed lines).

Figure 1 shows that several immune cells produce a variety of angiogenic and lymphangiogenic factors [43,44,51,123,137,147,149,156,157]. Immunologic and non-immunologic stimuli induce the release of VEGF-A from human mast cells [44,158,159,160]. These cells express different isoforms of VEGF-A (121, 165, 189 and 206) and their activation induces the release of VEGF-A [44]. Mast cells also express two isoforms of VEGF-B (167 and 186) and VEGF-C and VEGF-D. VEGFs induce mast cell chemotaxis in vitro [44] and in vivo [79] by binding to both VEGFR1 and VEGFR2. These cells also promote tumour growth by increasing the angiogenic supply, degradation of the extracellular matrix (ECM) and immunosuppression [161].

## 4. Mast Cells in the Immune Contexture of Cancer

Several studies have contributed to the characterization of the immune microenvironment of human gastric cancer. Figure 1 schematically illustrates the immune landscape of human gastric cancer. Several immune cells (M2 macrophages, TAM, mast cells, basophils, monocytes, PMN-MDSC, M-MDSC, Treg cells, Th2 cells, TAN, immature DCs and Th17/Tc17 cells), localized in human gastric cancer, release a wide spectrum of proinflammatory, angiogenic, lymphangiogenic and immunomodulatory mediators that play a protumorigenic role. Other immune cells (M1 macrophages, cytotoxic CD8^+^ T cells, NK cells, Th1 cells and mature DCs) and their mediators can play an anti-tumorigenic role in cancer. Eosinophils are component of the immune microenvironment that modulates tumour initiation and progression [162,166]. There are several bidirectional mast cell-eosinophil interactions in inflammatory disorders and cancer [167]. Increasing evidence indicates that eosinophils play an anti-tumorigenic role in different cancers [162,163,164]. The pro- (Tfh cells, type II NKT cells) or anti-tumorigenic role (γδ T cells, type I NKT cells and Th9 cells) of several immune cells have been demonstrated in other human cancers or are still under investigation in gastric cancer.

In the majority of tumours, such as thyroid [79,109], gastric [168,169,170,171], pancreas [78,172,173,174,175,176], bladder [177] and colorectal [178,179,180,181] cancers, hepatocellular carcinoma [182,183,184], Merkel cell carcinoma [75], Hodgkin’s [85,86,88] and non-Hodgkin’s lymphoma [87,89,92] and plasmacytoma [90,185], mast cells conferred poor prognosis. In breast cancer mast cells appear to play an antitumorigenic role [186,187,188]. These findings indicate that the contribution of mast cells to cancer is tumour dependent. 

Low mast cell density in perilesional stroma of invasive melanomas predicts poor prognosis [82], whereas, mast cell count was not correlated with survival in superficially invasive melanomas. Mast cells were pro-tumorigenic in the initial stages of prostate cancer but became dispensable at later stages [80,189]. A recent study examining a total of 9393 prostatectomy samples found that mast cell density was associated with better prognosis (i.e., distant metastasis-free survival) [190]. Peritumoral, but not intratumoral mast cell density, conferred a survival advantage in stage I non-small-cell lung cancer (NSCLC) but not in stage II [191]. The contributory role of mast cells in cancer varies according to the stage of tumorigenesis.

In prostate cancer, increased intratumoral mast cell density was associated with favourable prognosis [84]. Intratumoral mast cells inhibited tumour growth, whereas peritumoral mast cells stimulated human prostate cancer [76]. In NSCLC, mast cell in tumour islets was associated with a good prognosis [192,193], whereas only in stage I NSCLC increased peritumoral mast cells were conferred a survival advantage [191]. In pancreatic carcinoma, mast cell density in the intratumoral border zone but not the peritumoral or the intratumoral zone, was associated with disease progression [194]. The role of mast cells in melanoma depends on both the microlocalization of these cells [82] and the subtypes of tumour [195]. Mast cell density at the periphery of the tumours correlated with disease progression in both cutaneous T- and B-cell lymphoma [89]. Collectively, these findings indicate that the contribution of mast cells in tumours varies according to their microlocalization. 

In conclusion, the results of several studies indicate that the pro- or anti-tumorigenic role(s) of mast cells in different tumours is cancer specific, depends on the stage of tumorigenesis and on their microlocalization. It is possible that different subtypes of mast cells play a protective role whereas other types play a protumorigenic role. Single-cell mapping of peritumoral and intratumoral mast cells could help to elucidate the roles of different subsets of mast cells in the onset and progression of different tumours.

## 5. Mast Cells in the Immune Contexture of Human Gastric Cancer

Mast cells were first identified in small groups of Italian patients with gastric cancer more than 50 years ago [196,197]. Mast cell density was also found increased in Japanese patients with gastric cancer compared to macroscopically normal tissue [198]. Mast cells in gastric cancer were found to be chymase^+^ and it was suggested that patients with high number of mast cells had a poor prognosis [199]. *Helicobacter pylori (H. pylori)* is the etiologic agent of chronic gastritis and is recognized as a class 1 carcinogen [3]. Mast cells, eosinophils and basophils are increased in *H.pylori*-induced gastritis [200,201,202]. An increased density of mast cells was reported in patients with chronic gastritis [203]. Interestingly, elevated eosinophil density was found in the gastric cancer low-risk area, whereas in the high-risk area the eosinophil infiltrate was reduced. The authors speculated that eosinophils may promote or limit chronic inflammation and tumorigenesis depending on the surrounding immune environment.

Ribatti and collaborators highlighted the correlation between mast cells and angiogenesis in gastric cancer [204]. A correlation was also found between mast cell density and both Foxp3^+^ Treg cells and different stages of gastric cancer [205]. A correlation was also found between KIT^+^ mast cells and angiogenesis evaluated as microvascular density [169] and between tryptase^+^ mast cells and the number of metastatic lymph nodes in different stages of gastric cancer [168]. Mast cell tryptase is one of the proangiogenic factors stored and released by human mast cells [35,51,66,206]. Tryptase activates the protease-activated receptor-2 (PAR-2) on endothelial cells and a correlation was found between mast cell density and PAR-2 on endothelial cells in gastric cancer [207]. Based on the above findings it has been proposed that targeting tryptase could be a potential anti-angiogenic strategy in gastric cancer [208]. Ammendola and co-workers made an interesting observation looking at mast cells in bone metastases from gastric cancer patients [209]. They described the presence of mast cells near blood vessels in bone metastases from gastric cancer and found a correlation between mast cell density and microvascular density. The latter observation led to suggest that tryptase inhibitors or KIT tyrosine kinase inhibitors could represent a novel strategy to inhibit tumour-induced angiogenesis and osteoclastic bone resorption [210].

IL-17 is a pleiotropic cytokine [211] identified in several tumours including gastric cancer [212,213]. Although it has long been considered that the major source of IL-17 are CD4^+^ T lymphocytes (Th17 cells), this cytokine can be produced by several immune cells, including cytotoxic CD8^+^ T cells (Tc17), γδ T cells, NKT and NK cells, macrophages, granulocytes and mast cells [214,215,216]. It has been shown that activated mast cells are capable of expanding Th17 cells through the release of IL-1β [217]. In a study of gastric cancer patients, it was found that mast cells and to a lesser extent macrophages stained positively for IL-17 [218]. Furthermore, endothelial cells expressed IL-17 receptor (IL-17R) and intratumor mast cells IL-17^+^ were associated with worse overall survival. Recently, the prognostic value of IL-17 mRNA and IL-17A^+^ cells has been studied in two independent large cohorts of Chinese gastric cancer patients [171]. The overall survival was longer in the high intratumoral IL-17A^+^ cell group than in the low intratumoral IL-17A^+^ cell group. The authors also examined the immune contexture in different IL-17A mRNA expression status. High IL-17A mRNA expression was associated with high proportion of activated mast cells, NK cells and Tregs, while it was associated with low proportion of M2 macrophages and resting mast cells. Finally, it has been reported that activated mast cells release IL-17A which promoted the in vitro proliferation of gastric cancer cells [129]. 

**Figure 2:**
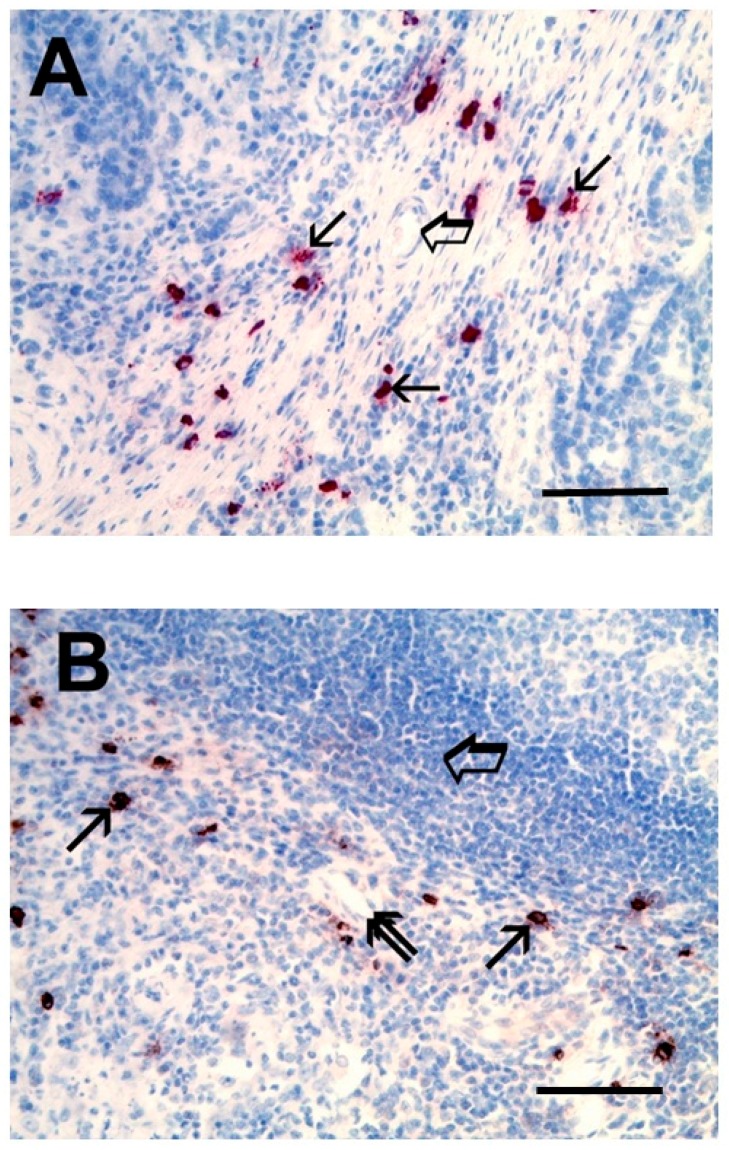
(**A**) Primary gastric cancer tissue immunostained with an anti-tryptase antibody demonstrates the presence of several mast cells in red (single arrow). Big arrow indicates a blood vessel with a red blood cell in its lumen (40 ×). (**B**) Metastatic lymph node from primary gastric cancer immunostained with an anti-tryptase antibody. Single arrows indicate red stained mast cells; the big arrow indicates a lymphocyte and the double arrow indicates a blood vessel (40 ×). Reprinted from Ammendola et al. (Int. J. Mol. Sci. 17: E1905, 2016). Bars: A and B = 100 μm.

The role of mast cells has also been started to be evaluated in metastatic lymph nodes of gastric cancer patients. Although mast cells are rarely found in normal lymph nodes, local mastocytosis was demonstrated in lymph node metastases from primary gastric cancer [219]. Figure 2A illustrates the localization of tryptase^+^ mast cells in primary gastric cancer. Interestingly, tryptase^+^ mast cells were also found in lymph node metastasis from primary gastric cancer (Figure 2B). The role of metastasis-associated mast cells is of great interest considering the contribution of these cells to lymphangiogenesis through the production of lymphangiogenic factors [44,220,221].

Recently, the spatial distribution of mast cells and vessels in peritumoral and intratumoral gastric cancer has been started to be investigated. It was found that tryptase^+^ chymase^+^ mast cells were preferentially located near the gastric glands and blood vessels [222]. In two large groups of patients with gastric cancer, peritumoral (area ≥ 2 cm from the tumour margin) and intratumoral (tumour centre area) mast cells were identified [171]. This study also examined the immune contexture of gastric cancer. CD4^+^ and CD8^+^ T cells, B cells, DCs, M0, M1 and M2 macrophages, monocytes, eosinophils, neutrophils, Tfh cells, Tregs, NK cells and plasma cells, in addition to mast cells, were found in the tumour microenvironment of gastric cancer [171]. The presence of mast cells and macrophages in gastric tumour microenvironment has been correlated to microvascular density [223]. The microlocalization of intratumoral, marginal, peritumoral and non-tumour issues of mast cells has been examined in gastric cancer patients [129]. These patients showed a higher mast cell infiltration in intratumoral tissues than marginal, peritumoral and non-tumour tissues. Moreover, as the cancer progressed from stage I to IV, the intratumoral mast cells increased, suggesting a possible protumorigenic role for these cells. It has been reported that mast cells accumulate in gastric cancer through the engagement of the chemokine receptor CXCR4 by CXCL12 produced by tumour cells [170].

Mast cells are immune sentinels in the surrounding microenvironment and rapidly perceive biochemical and immunological insults [39,67] through the engagement of a constellation of surface receptors [66]. These cells also express co-receptors for T-cells such as CD40 ligand (CD40L), tumour necrosis factor superfamily member 4 (OX40L), inducible costimulator ligand (ICOS-L). T cell immunoglobulin and mucin domain-containing protein 3 (TIM-3) and programmed death ligands (PD-L1 and PD-L2) [224,225,226]. The latter receptors are particularly relevant as immune checkpoint inhibitors (ICIs) [227,228]. Controversial results are reported about the impact of PD-L1 expression in gastric cancer [229,230]. Interestingly, intratumoral mast cells from gastric cancer constitutively expressed PD-L1 but not other molecules with immunosuppression potential such as CTLA-4 and ICOS [170]. TNF-α selectively induced the overexpression of PD-L1 on gastric mast cells. When mast cells from tumour and non-tumour tissues of gastric cancer patients were co-cultured with autologous peripheral blood CD3^+^ T cells, only tumour infiltrating mast cells inhibited T cell proliferation and IFN-γ production suggesting a specific immunosuppressive function. This hypothesis was extended in in vivo experiments using the NOD/SCID mice bearing SGC-7901-derived gastric cancer. In this model PD-L1 blocking antibody reduced gastric cancer progression. These important studies have identified a novel mechanism by which mast cells can promote tumorigenesis in gastric cancer and provide a rationale for the treatment of gastric cancer patients with immune checkpoint inhibitors (ICIs) targeting the PD-1/PD-L1 pathway.

## 6. Outstanding Questions and Conclusions

Gastric cancer is a heterogeneous disease [6,7,8,9] and several subtypes have been described anatomically [10], histologically [11] and genetically [12,14,15,16]. Several groups have identified tryptase^+^ and tryptase^+^ chymase^+^ mast cells in human gastric cancer patients in Europe [168,204,207,219,222,223] and in Asia [198,199,205,218]. Mast cell density in tumour microenvironment was associated with poor prognosis [129,168,170,198,199,218], tumour angiogenesis [169,198,199,204,219] and the formation of lymph node [168,219] and bone metastases [209]. These observations led to suggest that angiogenesis blockade could represent a promising target for the treatment of gastric cancer [207,210,231,232]. The results of several clinical trials indicate that anti-angiogenic agents improve overall survival, progression-free survival and disease control rate in gastric cancer [233]. Unfortunately, no studies thus far have identified a predictive biomarker to assist patient selection for benefit from anti-angiogenic agents. It would be interesting to verify whether mast cell density and/or activation in gastric tumours represent a biomarker of response to anti-angiogenic agents in these patients.

Mast cells are rarely found in normal lymph nodes. Figure 2B and elegant studies by Ammendola and collaborators have demonstrated that the density of mast cells is markedly increased in metastatic draining lymph nodes of gastric cancer patients [168,219]. This suggests that mast cells can migrate to tumour draining lymph nodes (TDLNs) where they can act as non-professional antigen presenting cells [234,235]. The mast cell contribution to the evolving microenvironment of TDLNs remains poorly understood. High-dimensional analysis, particularly single-cell RNA-seq, will be necessary to better characterize mast cells in TDLNs. 

Tumour cells evade host immune attack by expressing several immune checkpoints such as PD-1 and its ligands (PD-L1 and PD-L2) in TME. Monoclonal antibodies targeting the PD-1/PD-L1 pathway unleash anti-tumour immunity and have revolutionized the management of a wide spectrum of malignancies [236]. PD-L1 is overexpressed in up to 50% of gastric cancers [237,238] and a large number of clinical trials are evaluating the efficacy of mAbs anti-PD-1 (i.e., nivolumab, pembrolizumab) (Table S1) and anti-PD-L1 (i.e., atezolizumab, avelumab, durvalumab) (Table S2) as monotherapy or in combination with anti-CTLA-4 (i.e., ipilimumab) or targeted therapies in the management of advanced-stage gastric cancer. Human mast cells express PD-L1 and, to a lesser extent, PD-L2 [224,225,226]. An interesting task will be to investigate whether the expression of PD-L1 on mast cells is correlated with PD-L1^+^ cancer cells in the context of immunotherapy of gastric cancer.

As shown for tumour-associated macrophages (M1, M2, etc.) [101,102,103] and tumour-associated neutrophils (N1 and N2) [104,105,106], subpopulations of mast cells are recently begun to emerge [99,100] and could play different, even opposite effects in various types of tumours. Mast cells, like other immune cells, are endowed with phenotypic and functional plasticity depending on environmental factors [239] which may vary in composition in the different cancers [240,241]. The complex heterogeneity (spatial, temporal, intratumoral) of the TME adds a further layer of complexity. Simultaneous single-cell analysis of the immune contexture of TME of different subtypes of human gastric cancers characterized by genetic markers can greatly expand our knowledge of the role of mast cells in tumour initiation and progression.

All the above implies that clarification of the roles of subsets of mast cells in different human gastric cancers will demand studies of complexity beyond those assessing merely mast cell density, their microlocalization and the interactions with other immune cells. Therefore, many fundamental questions need to be addressed before understanding how mast cells play a protumorigenic role in gastric tumours.

## Supplementary Materials

Supplementary materials can be found at https://www.mdpi.com/1422-0067/20/9/2106/s1.

## Abbreviations

ANGPT	angiopoietin
BEC	blood endothelial cell
CD40	cluster of differentiation 40 protein
CD40L	cluster of differentiation 40 ligand
COX	cyclooxygenase
DC	dendritic cell
ECM	extracellular matrix
FLC	free light chain
ICI	immune checkpoint inhibitor
ICOS	inducible costimulator
ICOS-L	inducible costimulator ligand
IFN	interferon
IL	interleukin
KIT	stem cell factor receptor
LEC	lymphatic endothelial cell
mAb	monoclonal antibody
MDCS	myeloid-derived suppressor cell
MMP	matrix metalloproteinase
NK	natural killer cell
NKT	natural killer T cell
NSCLC	non-small-cell lung cancer
PAR	protease-activated receptor
PD-1	programmed death-1
PD-L1	programmed death ligand 1
PD-L2	programmed death ligand 2
PGE	prostaglandin E
PlGF	placental growth factor
PMN	polymorphonuclear leukocyte
SCF	stem cell factor
SCID	severe combined immunodeficiency
TAM	tumour-associated macrophage
TAMC	tumour-associated mast cell
TAN	tumour-associated neutrophils
TDLN	tumour draining lymph node
Tfh	T follicular helper cells
TIE	Tyrosine kinase with immunoglobulin-like and EGF-like domains
TIM-3	T cell immunoglobulin and mucin domain-containing protein 3
TME	tumour microenvironment
TNF	tumour necrosis factor
VEGF	Vascular endothelial growth factor
VEGFR	Vascular endothelial growth factor receptor
