# Supplementary material for: Mast Cells, Angiogenesis and Lymphangiogenesis in Human Gastric Cancer
Source: Int J Mol Sci. 2019 Apr 29;20(9):2106. doi: 10.3390/ijms20092106 (PMC6540185; doi:10.3390/ijms20092106)
Supplement: Supplementary file 1 [file ijms-20-02106-s001.pdf]

Table S1

Clinical Trials Evaluating the Effects of PD-1 Inhibitors in Gastric Cancer

NCT Number	PD-1 Inhibitor	Combination
NCT03784040	Nivolumab	Nivolumab + Ipilimumab
NCT03342417	Nivolumab	Nivolumab + Ipilimumab
NCT02872116	Nivolumab	Nivolumab + Ipilimumab<br>Nivolumab + Chemotherapy
NCT02999295	Nivolumab	Nivolumab + Ramucirumab
NCT03044613	Nivolumab	Nivolumab + Chemoradiation
NCT03704077	Nivolumab	Nivolumab + Relatlimab
NCT03647969	Nivolumab	Nivolumab + Ipilimumab
NCT03776487	Nivolumab	Nivolumab + Ipilimumab + Chemoradiation
NCT03409848	Nivolumab	Nivolumab + Ipilimumab + Trastuzumab
NCT03662659	Nivolumab	Nivolumab + Relatlimab + Chemotherapy
NCT03006705	Nivolumab	Nivolumab + Oxaliplatin
NCT02946671	Nivolumab	Nivolumab + Mogamulizumab
NCT03228667	Nivolumab	Nivolumab + Alt-803
NCT03841110	Nivolumab	Nivolumab + FT500 (NK)
NCT03228667	Pembrolizumab	Pembrolizumab + Alt-803
NCT03841110	Pembrolizumab	Pembrolizumab +FT500 (NK)
NCT03342937	Pembrolizumab	
NCT02901301	Pembrolizumab	Pembrolizumab + Oxaliplatin + Capecitabine
NCT03609359	Pembrolizumab	Pembrolizumab + Trastuzumab
NCT03849469	Pembrolizumab	Pembrolizumab + Lenvatinib
NCT02370498	Pembrolizumab	Pembrolizumab + XmAb22841
NCT02494583	Pembrolizumab	Pembrolizumab + Paclitaxel
NCT02335411	Pembrolizumab	Pembrolizumab
NCT02757391	Pembrolizumab	Pembrolizumab
NCT03797326	Pembrolizumab	Pembrolizumab + $CD8^+$ T Cell
NCT03122548	Pembrolizumab	Pembrolizumab + Lenvatinib
NCT02178722	Pembrolizumab	Pembrolizumab + CRS-207
NCT03043664	Pembrolizumab	Pembrolizumab + Epacadostat
NCT03675737	Pembrolizumab	Pembrolizumab + Lanreotide
NCT02943603	Pembrolizumab	Pembrolizumab + Chemotherapy

NCT03221426	Pembrolizumab	Pembrolizumab + m FOLFOX6
NCT02318901	Pembrolizumab	Pembrolizumab + Chemotherapy
NCT03095781	Pembrolizumab	Pembrolizumab
NCT02730546	Pembrolizumab	Pembrolizumab + XL888
NCT030119588	Pembrolizumab	Pembrolizumab + Chemotherapy + Radiation
NCT03488667	Pembrolizumab	Pembrolizumab
NCT02563548	Pembrolizumab	Pembrolizumab + m FOLFOX
NCT02346955	Pembrolizumab	Pembrolizumab + Hyaluronidase
NCT03615326	Pembrolizumab	Pembrolizumab + CM-24
NCT03196232	Pembrolizumab	Pembrolizumab + Trastuzumab
NCT03724851	Pembrolizumab	Pembrolizumab + Epacadostat
NCT02954536	Pembrolizumab	Pembrolizumab + Vactosertib
NCT02918162	Pembrolizumab	Pembrolizumab + Trastuzumab
NCT03382600	Pembrolizumab	Pembrolizumab + Chemotherapy
NCT02689284	Pembrolizumab	Pembrolizumab + Oxaliplatin
NCT03064490	Pembrolizumab	Pembrolizumab + Margetuximab
		Pembrolizumab + Radiotherapy + Chemotherapy

Table S2

Clinical Trials Evaluating the Effects of PD-L1 Inhibitors in Gastric Cancer

NTC Number	PD-L1 Inhibitor	Combination
NCT03841110	Atezolizumab	Atezolizumab +FT500 (NK)
NCT03421288	Atezolizumab	Atezolizumab + FLOT
NCT03448835	Atezolizumab	Atezolizumab + Capecitabine + Oxaliplatin + Docetaxel
NCT03650348	Atezolizumab	Atezolizumab + PRS-343
NCT03818997	Atezolizumab	Atezolizumab
NCT03829501	Atezolizumab	Atezolizumab + KY1044
NCT03170960	Atezolizumab	Atezolizumab + Cabozantinib
NCT03399071	Atezolizumab	Atezolizumab + Chemotherapy
NCT02876510	Atezolizumab	Atezolizumab + $CD8^{+}$ T Cells
NCT03841110	Atezolizumab	Atezolizumab + NK Cells
NCT03228667	Atezolizumab	Atezolizumab + ALT-803
NCT02625610	Avelumab	Avelumab
NCT02625623	Avelumab	Avelumab
NCT01943461	Avelumab	Avelumab
NCT03475953	Avelumab	Avelumab + Regorafenib
NCT03399071	Avelumab	Avelumab + Chemotherapy
NCT02554812	Avelumab	Avelumab + Utomilumab
NCT03228667	Avelumab	Avelumab + ALT-803
NCT03579784	Durvalumab	Durvalumab + Olaparib + Paclitaxel
NCT03751761	Durvalumab	Durvalumab + Tremelimumab
NCT02734004	Durvalumab	Durvalumab + Olaparib
NCT03539822	Durvalumab	Durvalumab + Cabozantinib
NCT02572687	Durvalumab	Durvalumab + Ramucirumab
NCT02658214	Durvalumab	Durvalumab + Tremelimumab
